# Development of Mucoadhesive Buccal Film for Rizatriptan: In Vitro and In Vivo Evaluation

**DOI:** 10.3390/pharmaceutics13050728

**Published:** 2021-05-15

**Authors:** Anroop B. Nair, Jigar Shah, Shery Jacob, Bandar E. Al-Dhubiab, Vimal Patel, Nagaraja Sreeharsha, Pottathil Shinu

**Affiliations:** 1 Department of Pharmaceutical Sciences, College of Clinical Pharmacy, King Faisal University, Al-Ahsa 31982, Saudi Arabia; baldhubiab@kfu.edu.sa (B.E.A.-D.); sharsha@kfu.edu.sa (N.S.); 2 Department of Pharmaceutics, Institute of Pharmacy, Nirma University, Ahmedabad 382481, India; jigsh12@gmail.com (J.S.); email2vimal.patel@gmail.com (V.P.); 3 Department of Pharmaceutical Sciences, College of Pharmacy, Gulf Medical University, Ajman 4184, United Arab Emirates; sheryjacob6876@gmail.com; 4 Department of Pharmaceutics, Vidya Siri College of Pharmacy, Off Sarjapura Road, Bangalore 560035, India; 5 Department of Biomedical Sciences, College of Clinical Pharmacy, King Faisal University, Al-Ahsa 31982, Saudi Arabia; spottathail@kfu.edu.sa

**Keywords:** migraine, Proloc, Eudragit, physicomechanical, release, in vivo, pharmacokinetics

## Abstract

The reduced therapeutic efficacy of rizatriptan in migraine treatment is primarily due to low oral bioavailability and extensive first pass metabolism. The purpose of this investigation was to optimize the thin mucoadhesive buccal film of rizatriptan and assess the practicability of its development as a potential substitute for conventional migraine treatment. Buccal films (FR1–FR10) were fabricated by a conventional solvent casting method utilizing a combination of polymers (Proloc, hydroxypropyl methylcellulose and Eudragit RS 100). Drug-loaded buccal films (F1–F4) were examined for mechanical, mucoadhesive, swelling and release characteristics. In vivo pharmacokinetics parameters of selected buccal film (F1) in rabbits were compared to oral administration. Films F1–F4 displayed optimal physicomechanical properties including mucoadhesive strength, which can prolong the buccal residence time. A biphasic, complete and higher drug release was seen in films F1 and F4, which followed Weibull model kinetics. The optimized film, F1, exhibited significantly higher (*p* < 0.005) rizatriptan buccal flux (71.94 ± 8.26 µg/cm^2^/h) with a short lag time. Film features suggested the drug particles were in an amorphous form, compatible with the polymers used and had an appropriate surface morphology suitable for buccal application. Pharmacokinetic data indicated a significantly higher rizatriptan plasma level (*p* < 0.005) and C_max_ (*p* < 0.0001) upon buccal film application as compared to oral solution. The observed AUC_0–12h_ (994.86 ± 95.79 ng.h/mL) in buccal treatment was two-fold higher (*p* < 0.0001) than the control, and the relative bioavailability judged was 245%. This investigation demonstrates the prospective of buccal films as a viable and alternative approach for effective rizatriptan delivery.

## 1. Introduction

The International Headache Society defines migraine as a recurrent primary neurological disorder characterized by headache with or without aura. Various clinical symptoms and neurological disturbances reported during all phases of migraine are intense and complex. As per the revised version of global burden of disease study, migraine remains the third major cause of disability, affecting both males and females under the age of 50 years [1]. Many epidemiological studies have documented its public health, socio-economic and psychological impacts on individuals and society [2]. The preventive medications that are used to diminish the incidence or harshness of migraine attacks include antiepileptics, antidepressants and beta blockers. On the other hand, drug categories including triptans, corticosteroids, NSAIDs and analgesics are indicated in acute migraine attacks [3]. The first-line acute therapy for patients having moderate to severe migraine attacks include the triptans and serotonin (5-hydroxytryptamine [5-HT]) subtype _1B/1D/(1F)_ receptor agonists [4]. Their mechanisms of action mediated through the activation of 5-HT_1B_ include cranial vasoconstriction, the inhibition of calcitonin gene-related peptide release and antinociceptive modulation [5]. All triptans are considered to be more effective and safe drugs among most migraine patients [6]. Though they possess similar molecular structures, individual triptans have unique pharmacokinetic and pharmacodynamic profile. Rizatriptan, a BCS class III and second generation oral triptan with high potency, quick onset of action, nano-molar affinity and highly specific and selective 5-HT_1B/1D_ agonists has been considered in the treatment of acute migraine attacks [3]. The recommended dose of rizatriptan in acute migraine attacks is between 5 and 10 mg [7]. Indeed, this drug possess better clinical characteristics like superior efficacy and higher safety and tolerability than other triptans [8]. However, the clinical efficacy of this active pharmaceutical ingredient is still low, primarily due to its low oral bioavailability (~20%) and extensive first-pass metabolism [9]. In this context, developing an alternative drug delivery system or delivering rizatriptan through another route is likely to be advantageous. Attempts have been made to improve the clinical efficacy of this drug by delivering it through the oral, nasal and transdermal routes using different approaches [10]. A literature survey reported the utilization of various drug delivery systems such as orally disintegrating tablets, pulsatile capsules, thermoreversible nasal gel, microspheres, nanoemulsion, nanoparticles and buccal film/patches to improve the bioavailability of rizatriptan [10,11].

Due to excellent accessibility, the absence of a first pass effect, excellent blood supply, safety and patient compliance, mucoadhesive buccal therapy has become popular and received wider acceptance during the last few decades [12]. In addition, the buccal route is most suitable for the non-invasive delivery of pharmaceutical actives generally used in acute therapy [13]. Buccal therapy has attained commercial status, and marketed products include soluble buccal films of buprenorphine hydrochloride (Belbuca), fentanyl buccal soluble film (Onsolis), fentanyl lozenges (Actiq), fentanyl buccal tablets (Fentora) and a combination of buprenorphine and naloxone (Bunavail) as well as the lidocaine patch (Dentipatch) [14]. The highly perfused oral mucosal membrane can provide a rapid permeation of drug molecules with low bioavailability as well as a short half-life into the blood circulation to deliver its therapeutic effect. Further, the buccal film can transport the drug in a steady and controlled manner and is therefore a better alternative for the oral route [15,16]. It also addresses the major limitations associated with oral therapy such as enzymatic degradation, variable drug absorption along the gastrointestinal tract and extensive hepatic biotransformation. On the other hand, the buccal film formulations can be easily scaled up because of the adaptable and feasible nature of film manufacturing processes such as solvent casting and hot melt extrusion techniques. Advancement in 3D printing technology will also help in accommodating adequate doses of drugs in buccal films with limited dimensions [17]. Moreover, the buccal films are prepared using generally regarded as safe excipients and, hence, could be a suitable drug delivery system for the pediatric population [14]. Furthermore, the physicochemical properties of rizatriptan such as a low molecular weight (269.34 Da), good log *p* (1.4) and low dose (10 mg) are all ideal for delivery through the buccal cavity. Upon consideration of the physicochemical properties and biopharmaceutical concerns of rizatriptan, it was decided to deliver it through the oral mucosal route, employing buccal film. Ideally, the clinical condition of a migraine episode requires rapid onset of action in addition to extended duration of action, which is achievable with buccal film. Hence, the purpose of the present investigation was to design, develop and optimize a mucoadhesive, thin buccal film of rizatriptan and evaluate its potentiality as an alternate therapy for migraine.

A wide range of polymers have been used in the development of mucoadhesive formulations [18,19,20,21,22]. In the current study, mucoadhesive buccal thin films were formulated using Proloc, hydroxypropyl methylcellulose (HPMC) and Eudragit RS 100 polymers. The selection criteria were based on the renowned film forming, mucoadhesive and release-modifying properties demonstrated by these polymers in various studies [13,23,24]. Further, based on extensive documentary evidence, propylene glycol and polyethylene glycol 200 were included as plasticizers [25], and Tween 80 as a solubilizing agent [26], for the development of buccal films. All the developed buccal films were examined for mechanical, mucoadhesive, swelling and release characteristics. The in vivo pharmacokinetics parameters of selected buccal film (F1) in rabbits were compared with oral therapy.

## 2. Materials and Methods

### 2.1. Materials

Rizatriptan benzoate (Emcure Pharmaceuticals, Ahmedabad, India), Proloc 15 (Henkel Corporation, Rocky Hill, CT, USA), HPMC F4M, HPMC K4M and HPMC K100M (Colorcon Limited, Goa, India) were obtained as a gift. Tween 80, propylene glycol and polyethylene glycol 200 (PEG 200) were procured (Sigma Aldrich, St. Louis, MO, USA). All other chemicals and reagents utilized in this research were commercially obtained from local traders with the highest quality.

### 2.2. High-Performance Liquid Chromatography (HPLC)

The quantification of rizatriptan in individual samples was done by HPLC (Shimadzu, Tokyo, Japan). A combination of solvent mixture constituted of methanol and water at a specific ratio (30:70) adjusted to pH 3 with orthophosphoric acid was used for the separation of rizatriptan with the aid of a monolithic C18 column (Chromolith^®^ Speed Rod). The uniform flow rate of the mobile phase was 1 mL/min, and the elution of the drug was monitored at 227 nm [27]. The analytical method validation studies were performed to assess sensitivity, selectivity, linearity, precision, accuracy, protein precipitation and ruggedness. The validated method demonstrated linearity in the concentration range between 5 and 1200 ng/mL (r^2^ = 0.9891).

### 2.3. Formulation of Films

Placebo buccal films (FR1–FR10) were formulated employing a typical solvent casting method. The formulation ingredients used in fabricating the films are summarized in Table 1. Briefly, Proloc 15 and HPMC were evenly dispersed in water under constant stirring (400 rpm), employing a magnetic stirrer (MS–4, Deepali United, Ahmedabad, India). Similarly, a dispersion of Eudragit RS 100 was made using ethyl alcohol (70% *v*/*v*) into which a combination of Proloc 15 and HPMC was uniformly mixed by means of continuous stirring. Next, a solvent combination consisting of PEG 200, propylene glycol and Tween 80 was included to the above dispersion and vortexed to get a homogeneous mixture. The dispersion (10 mL) was poured into separate petri dishes (90 mm diameter) and allowed to dry at 40 °C in a hot air oven. The characteristics of the developed placebo films from different batches (FR1–FR10) are given in Table 1.

### 2.4. Formulation of Drug-Loaded Films

Films (FR3–FR5 and FR9) were selected due to the advantages mentioned in Table 1 and combined with rizatriptan to create drug-loaded matrix films (F1–F4; Table 2) by following the same procedure as mentioned in the formulation of the films. The drug loading was done by adding 10 mL of the dispersion (containing 640 mg of rizatriptan; 6.4% *w*/*v*), casting on separate petri dishes (9 cm diameter; area 63.6 cm^2^) and drying to obtain 10 mg/cm^2^ (Table 2). A backing membrane made of a particular concentration of ethyl cellulose (5% *w*/*v*) and plasticizer, dibutyl phthalate (2% *v*/*v*), was affixed to the rizatriptan-containing film with the help of an adhesive polymer, polyvinyl pyrrolidone (5% *w*/*v*) [28].

### 2.5. Characterization of Buccal Films

Prepared, drug-loaded matrix films (F1–F4) were evaluated for different properties such as texture, crack, flexibility and homogeneity. The external features of the films such as softness and stickiness were subjectively examined by physical contact with developed films.

#### 2.5.1. Thickness and pH

Th thickness of the prepared films (F1–F4) at five diverse locations was measured using a digital micrometer (MDH-25M, Mitutoyo, Kawasaki, Japan). The surface pH was measured by randomly selecting three films having a surface area of 1 cm^2^ from each prepared batch and permitting them to swell in 5 mL of distilled water for 30 min [29]. The pH was determined using a Thermo Fischer Benchtop pH meter.

#### 2.5.2. Drug Content

Each buccal film measuring 1 cm^2^ of surface area were cut from different sites, immersed in a solvent system consisting of methanol–water and stirred in a thermostatically controlled (37 ± 1 °C) water bath for 6 h. The drug extracted into the solvent was filtered and analyzed using HPLC.

#### 2.5.3. Folding Endurance

Folding endurance was manually determined by repeated folding at the same axis using a film having a specified surface area of 4 cm^2^. The value was determined by counting the number of folds the film tolerated before tearing.

#### 2.5.4. Mucoadhesive Strength

Th mucoadhesive strength of films (F1–F4) was determined with a texture analyzer (Stable Micro Systems, Surrey, UK), employing rabbit buccal mucosa as substrate. Briefly, the buccal membrane was affixed onto the stationary stage, while the film with a suitable size of 1 cm^2^ was attached to the probe of the analyzer. The tissue membrane was wetted using simulated saliva. The movable probe was slid down gradually until the probe made contact with the mucus membrane and then remained for 1 min. The parameters used in our earlier study were followed while measuring the mucoadhesive strength [30].

#### 2.5.5. Percent Hydration

The swelling characteristic of the prepared films (F1–F4) was measured as percentage hydration. Briefly, films with specific dimensions (1 cm × 1 cm) (F1-F4) were accurately weighed (W_1_) and kept on a steel mesh. The film, along with mesh, was dipped in simulated saliva (10 mL) maintained at 37 ± 1 °C. The mesh was taken out from the saliva medium at various time intervals, and the film was wiped and reweighed (W_2_). The percentage hydration was determined using the equation in the literature [31].

### 2.6. Fourier Transform Infrared (FTIR)

Possible drug–excipient interaction was assessed by recording the spectra of the rizatriptan, physical mixture and the rizatriptan buccal film (F1) using a FTIR spectrometer (Jasco, Tokyo, Japan). Disc samples were prepared by intimately mixing the drug with KBr at a ratio of 1:5 by compression, employing a hydraulic press. The scanning of discs was carried out in the range of 400–4000 cm^−1^, and the peaks in the spectra were compared.

### 2.7. Differential Scanning Calorimetry

The thermograms of rizatriptan, buccal film (F1) and control film were obtained by differential scanning calorimeter (DSC; Shimadzu, Kyoto, Japan). Accurately weighed test samples (5 mg) were placed in individual aluminum pans and non-hermetically crimp sealed with aluminum covers. Samples were scanned at temperature ranges between 50 and 300 °C under a uniform heating rate (10 °C/min) with nitrogen gas flow.

### 2.8. Scanning Electron Microscopy

The surface morphology of optimized rizatriptan film (F1) was investigated with scanning electron microscopy (SEM). The micrograph of the film was captured on a Nova NanoSEM 450 (FEI Ltd., Hillsboro, OR, USA) equipped with a large field detector. The film was mounted using silver electrical tape and sputter coated (SCD005 Baltek Sputter Coater, Baltec, AG, Balzers, Liechtenstein, Germany) with gold in the presence of argon gas under reduced pressure [32].

### 2.9. Drug Release

The drug release from developed films F1–F4 was evaluated by means of the paddle-over-disc method, utilizing a USP Type II apparatus (Electrolab TDC 50, Mumbai, India) [33]. Films were chosen from each batch, and each individual film having particular dimensions (2 cm × 1 cm) was held to a glass slide. The entire assembly was kept at the bottom of the dissolution vessel in such a manner that the drug-entrapped surface could release it toward the dissolution medium (900 mL of simulated saliva), set at a temperature of 37 ± 0.5 °C. The composition of simulated saliva utilized in the study consisted of 12 mM of potassium di hydrogen phosphate, 40 mM of sodium chloride and 1.5 mM of calcium chloride, and the pH was adjusted to 6.8 using sodium hydroxide [34]. The paddle was rotated at 50 rpm and aliquot volumes of the samples were withdrawn, filtered using a syringe membrane filter having a pore size of 0.2 μm (Millipore, Bedford, MA, USA) and readily estimated by HPLC. The correlation coefficient (r^2^) was used to indicate the type of release mechanism from the buccal film by fitting the release data into widely used mathematical release kinetics models for films such as the zero order model, first order model, Higuchi model, Korsmeyer–Peppas model and Weibull model [35], utilizing kinetic software (Kinetics DS 3.0 rev 2010, SourceForge, Slashdot Media, San Diego, CA, USA).

### 2.10. Ex Vivo Permeation

The transmucosal permeation flux of rizatriptan from the selected buccal films (F1 and F4) and control (solution) was determined using Franz diffusion cells set at 37 ± 0.5 °C [13]. The buccal mucosal membrane of a rabbit was held between the donor and receiver chambers and had an active surface area of 0.64 cm^2^. Rizatriptan-containing films were punched to a specific size (0.6 cm^2^) or a control (0.6 mg) and placed on the surface of mucosa. The lower receiver chamber was filled with simulated saliva (5 mL) and stirred at 50 rpm. Aliquots (1 mL) of receiver fluid were withdrawn and replaced with the same fluid. The samples withdrawn were subsequently assayed by HPLC. The flux values were determined by measuring the slope of the individual permeation data plotted between the quantities of rizatriptan transported versus time [36]. For each formulation, a permeation study was carried out six times (*n* = 6), and all data represented mean ± standard deviation.

### 2.11. In-Vivo Evaluation

Male rabbits (2.5–3 kg) were kept separately in standard cages for 24 h in a well-ventilated animal house maintained at a room temperature of 25 °C. The animals (*n* = 12) were divided into two groups, each comprising six rabbits. For group 1 (treated group), optimized rizatriptan buccal film (F1) was applied, while in group II (control group), an equivalent dose of rizatriptan as an oral solution was administered. All experimental procedures were carried out according to the Institutional Animal Ethics Committee guidelines (IAEC/IP/PCEU/2019/094). Ketamine (40 mg/kg) and xylazine (5 mg/kg) were used to anesthetize rabbits [37]. A specific size of film (1 cm × 1 cm) constituting 10 mg of rizatriptan (for comparison) was moistened with a few drops of water (30 µL) and applied with mild force to the buccal area of the rabbits. A solution of rizatriptan (1 mL) equivalent to 10 mg was administered perorally to the control group using intragastric gavage. Blood samples (500 µL) were collected from the marginal veins of the rabbits at 0.5, 1, 1.5, 2, 4, 8 and 12 h. Proteins from these samples were subsequently precipitated by treatment with an equivalent ratio of 2- propanol and acetonitrile. The precipitated samples were then centrifuged (1789× *g* for 10 min), decanted to separate the supernatant layer and analyzed.

### 2.12. Data Analysis

The statistical analysis was carried out by GraphPad Prism 6 (Graph-Pad Software, Inc., La Jolla, CA, USA). The difference in *p* < 0.05 was identified as statistical significance.

## 3. Results and Discussion

### 3.1. Formulation of Buccal Films

An ideal mucoadhesive buccal film should be soft, flexible, compact, mechanically strong and possess adequate mucoadhesive strength. A combination of Proloc 15, HPMCs and Eudragit RS 100 was selected to obtain firm, compact and thin mucoadhesive buccal film based on our previous studies [31,38,39]. Preliminary investigations were carried out by preparing 10 placebo films (FR1–F10) with Proloc, 3 HPMCs (F4M, K100M and K4M) and Eudragit RS 100, as given in Table 1. Proloc exhibits both mucoadhesive as well as film-forming characteristics, which make it easily combined with other polymers to fabricate suitable mucoadhesive films. Therefore, different concentrations of Proloc (4–10% *w*/*v*) were used in combination with mucoadhesive HPMCs (2–6% *w*/*v*) and film-forming Eudragit RS 100 polymer (15% *w*/*v*). The selected HPMC polymers differed with viscosity, hydrophilicity, molecular mass or degree of substitution. Further, inclusion of plasticizer (PG; 2.5% *w*/*v*) facilitated more flexibility, peelability and homogeneity in the film. Wetting agent (Tween 80; 0.5% *w*/*v*) and water miscible solvent (PEG 200; 2.5% *w*/*v*) were incorporated to improve the mechanical strength as well as the drug release from drug loaded films. The film properties of placebo films are shown in Table 3. Appropriate film properties expected from the buccal film such as softness, peelability, non-tackiness, homogeneity and mechanical strength was met by formulations FR3, FR4, FR5 and FR9. Therefore, these formulations were chosen to incorporate rizatriptan, and films F1, F2, F3 and F4 were developed (Table 2). The drug level in the films was fixed at 6.4% *w*/*v* to obtain 10 mg/cm^2^ of rizatriptan.

### 3.2. Film Characteristics

Homogeneity of the film is a key parameter to establish the uniform distribution of the drug as well as predictable drug release from it [13]. The average thicknesses in films F1–F4 were about 1.02 mm to 1.32 mm (Table 3), signifying thin film that would be beneficial because it causes minimum discomfort to patients. Tissue irritation can be reduced if the pH difference between the applied film and the buccal mucosal surface is less. The pH of buccal film was examined to establish its appropriateness for buccal use, thereby avoiding any sort of sensitivity or allergic reactions. The pH of films F1–F4 was found to be almost neutral (pH 6.9–7.2) and near to the buccal pH 6.4 [40].

The folding endurance values were utilized to examine the pliability and durability of the various prepared buccal films. Data from Table 3 indicates that buccal films from all batches (F1–F4) retained high endurance values (>250) and were comparable. The highest folding endurance value (~305) was shown in film F3. This might be due to the complexity imparted to the film by the incorporation of highly viscous HPMC K100M and a greater concentration of Proloc (8% *w*/*v*). The higher folding endurance value was significant because mechanically strong film would resist tearing as well as detachment of the film from the site during application.

Uniformity of content is a critical pharmaceutical quality control criterion that is generally assessed to ensure drug availability in pharmaceutical products. The data displayed in Table 3 signifies higher drug content > 94% in films F1–F4. The consistent values among various formulations signified that the variation in polymer composition did not influence rizatriptan content.

Mucoadhesion is a key factor responsible for successful buccal therapy because inadequate mucoadhesion could lead to the displacement of film from the site of application. The data in Table 3 demonstrates that films F1–F4 possessed adequate mucoadhesive strength (>6.5 N), concurrently contributed by Proloc 15 and HPMC. The mucoadhesive property exhibited by Proloc 15 could be due to the carbomer present in it [39]. Higher mucoadhesive strength (approximately 7.3 N) was observed in film F3, prepared with 8% *w*/*v* of Proloc, than films made with low concentrations, which substantiated previous studies [13]. Indeed, the good mucoadhesive strength displayed by films favored their retention in the buccal mucosa for long durations.

**Figure 1:**
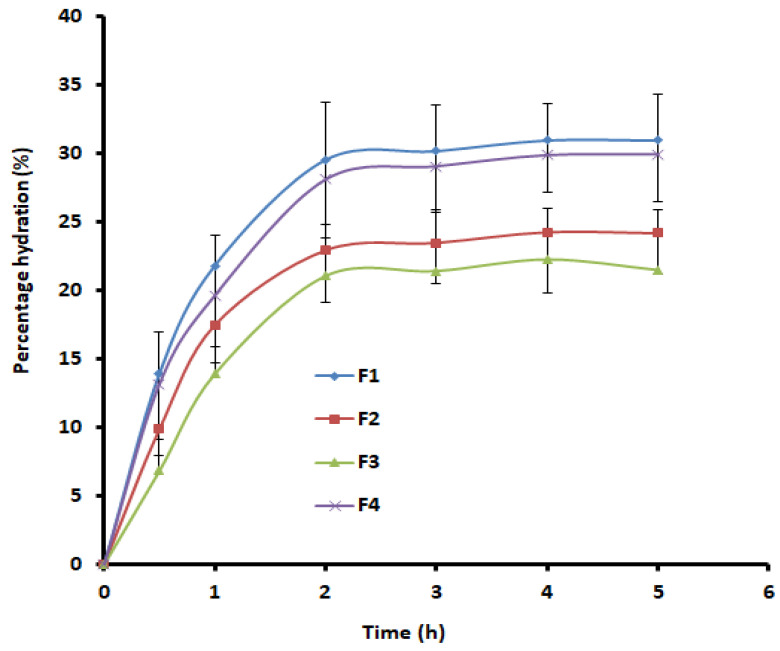
Comparison of percentage hydration of buccal films (F1–F4). Data represented are mean ± SD (*n* = 6).

Swelling due to water uptake allows initially stretched, twisted or entangled bioadhesive polymers to relax, resulting in rapid disentanglement of individual polymer chains and generating a macromolecular network of a specific size that increases the porosity of the film and initiates drug release [41]. However, too-extensive swelling may lead to discomfort for patients. Typically, film hydration depends on the type and physicochemical nature of the film formers and their composition [42]. It is evident from Figure 1 that the hydration values in films F1 and F4 were slightly higher and comparable, while they were low in films F2 and F3. The minor enhancement in hydration in films F1 and F4 could be due to the incorporation of more hydrophilic HPMC F4M. Indeed, the percentage swelling rate was rapid in all films (F1–F4), as evidenced by a sharp curve in the initial hour that continued to improve further until 2 h. Thereafter, the percentage hydration remained steady, suggesting there was no further swelling. Rapid hydration in 2 h (20–30%) suggested that the prepared films were capable of swelling and providing adequate mucoadhesion during application. Indeed, a relationship exists between the swelling index and mucoadhesive strength, wherein the mucoadhesion increases with the degree of hydration until the point where hydration leads to an abrupt drop in adhesive strength due to disentanglement at the polymer–tissue interface [28,43]. Polymer swelling ensures that the polymer chains uncoil and promote hydrogen bonding and/or electrostatic interaction between polymer and mucin [41,44]. Moreover, the prompt hydration detected might be due to the hydrophilic properties of the HPMC and Proloc 15.

### 3.3. FTIR

**Figure 2:**
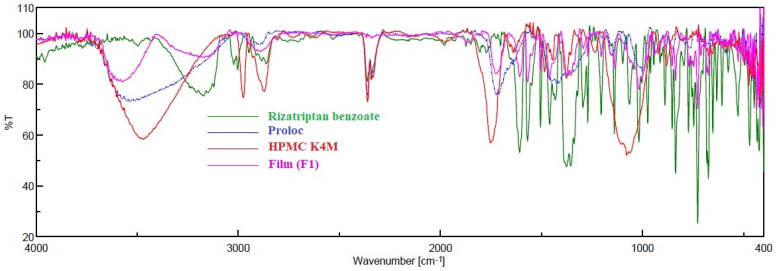
FTIR spectra of rizatriptan, Proloc, HPMC F4M and drug-loaded buccal film (F1).

During formulation and development, drug–excipient compatibility studies are typically performed by FTIR. The additives included in the formulation can potentially interact with active pharmaceutical ingredients that can cause molecular transformation, which ultimately affects the stability of the product [45]. Figure 2 represents the FTIR spectra of rizatriptan benzoate, Proloc, HPMC F4M and a selected buccal film (F1). The predominant peaks relate to the main functional groups of pure rizatriptan benzoate that showed characteristic spectral peak positions at 1609 cm^−1^, representing C=C stretching vibration in aromatic rings, 1370 cm^−1^ refers to C-N stretching in tertiary amines and 1290 cm^−1^ corresponds to the C-O stretching vibrations of carboxylic acid [46]. The spectra of optimized buccal film (F1) also showed all essential peaks of the pure drug and no evidence of significant peak shifts. Therefore, it can confirm that there are no compatibility issues between the drug and other excipients used in the film.

### 3.4. DSC

**Figure 3:**
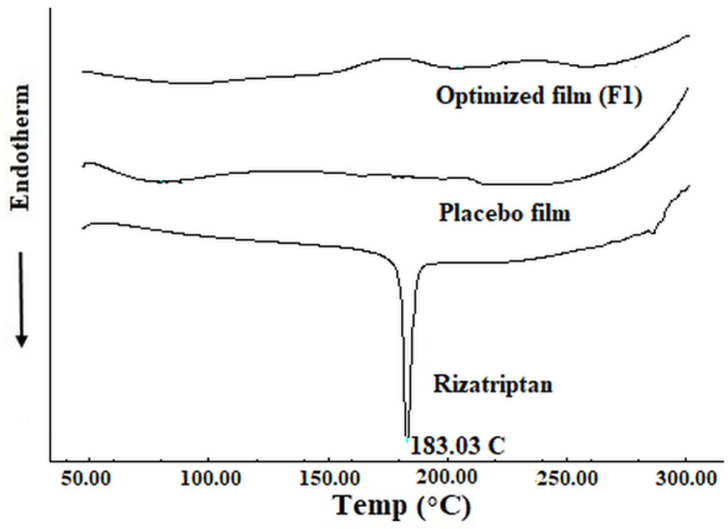
Differential scanning calorimetric curves of rizatriptan, drug loaded film (F1) and placebo film.

Calorimetry is a principal technique used in determining the thermal behavior of constituents in film. Therefore, solid-state transformations such as crystallization and melting, indicated by exothermic and endothermic peaks, were evaluated within the optimized film. The thermal scans of rizatriptan, optimized film (F1) and placebo film are depicted in Figure 3. The crystalline form of rizatriptan was displayed by a prominent, endothermic melting peak at 183.03 °C [46]. In the case of film F1, no specific melting peak was seen at 183.03 °C. The absence of a peak in the thermogram signifies that the drug was in an amorphous state in the film, reduced its crystallinity during film formation or that the drug level may have been lower than the detection limit. The thermogram of the placebo film did not show any characteristic peaks.

### 3.5. SEM

**Figure 4:**
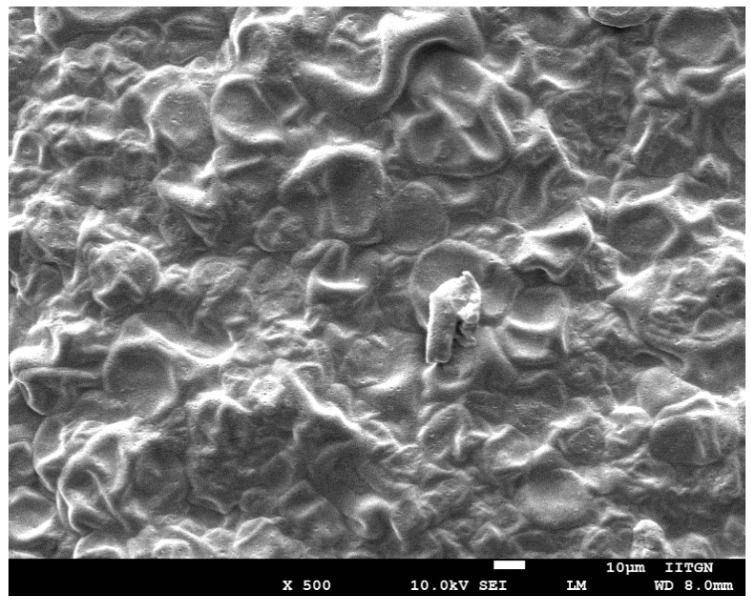
Scanning electron microscopy image of drug-loaded film (F1) at 500× magnification.

SEM was performed to get an insight into the topography, texture and morphology of sectioned film surfaces. It is clear from Figure 4 that the optimized film (F1) appears to be marginally rough, has an uneven texture and is tortuous in nature. However, no visible pores or cracks were seen on the representative micrograph, which is necessary to control the hydration and release of drug molecules [47]. The absence of drug crystals in the micrographs signified that the drug particles were homogeneously dispersed within the polymer matrix. Indeed, the external characteristics of the drug-entrapped film had appropriate surface morphology and were therefore suitable for buccal application.

### 3.6. Drug Release

**Figure 5:**
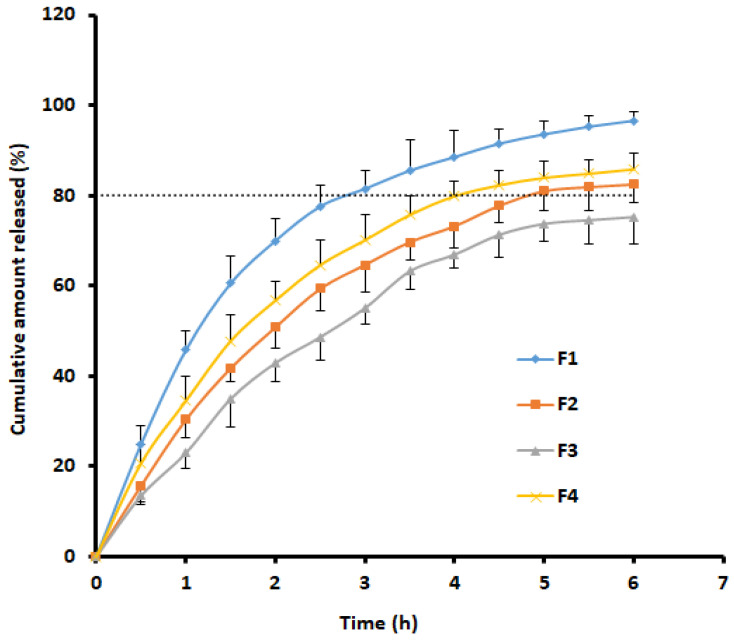
Cumulative percentage release of rizatriptan from buccal films (F1–F4). Data represented are mean ± SD (*n* = 6).

In vitro drug release studies are important to learn about the liberation of therapeutic actives from the film to the buccal mucosa and subsequent permeation through this biological membrane. The correlation among in vitro release data and in vivo absorption is also demonstrated [33,48]. Further, it is a well-known fact that drug delivery from a formulation is primarily dictated by the properties of the drugs and polymers [49]. The effect of the polymer composition (Proloc and three HPMCs) on the release of rizatriptan from films F1–F4 was determined and illustrated in Figure 5. A similar trend in drug release was observed in all the prepared films, as observed in Figure 5. It seemed the drug release was biphasic, as evidenced by a higher drug release rate in the initial two hours (the amount of drug released was 40–70%). This type of release profile is anticipated in buccal delivery, as greater drug release in the initial period will ensure adequate drug availability on the mucosal surface for absorption. It is also apparent from the profiles that drug release was relatively higher in film F1 and was nearly complete in 6 h. Followed by F1, the drug release decreased as F4 > F2 > F3, indicating the film composition influenced the rizatriptan release. The higher drug release observed with film F1 could be due to the presence of more hydrophilic HPMC F4M, wherein the drug release is usually due to swelling [50]), which also supports the hydration data (Figure 1). Film F4 stood second, which has lower hydrophilic HPMC (K4M) than F4M. The release of rizatriptan from films F1 and F4 followed Weibull model kinetics. As the drug release was relatively higher in films F1 and F4, they were chosen for additional ex vivo investigations.

### 3.7. Ex Vivo Permeation

**Figure 6:**
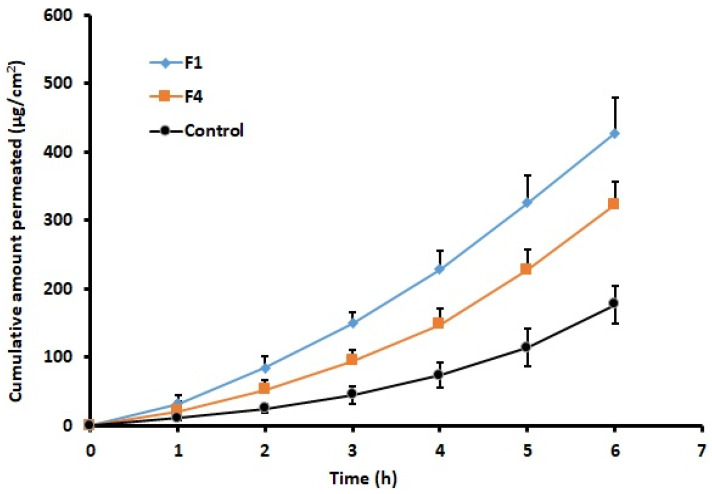
Ex vivo permeation of rizatriptan across rabbit buccal mucosa from buccal films (F1 and F4) and control. Data represented are mean ± SD (*n* = 6).

Ex vivo permeation experiments are generally performed to get insight about the absorption kinetics of drugs through the biological membranes [51]. In general, drug transport through any membrane is regulated by the nature of the drug molecules and the underlying physiology of the biological barriers. Figure 6 illustrates the quantity of rizatriptan transported across the rabbit buccal mucosa from films (F1 and F4) and the control. Indeed, the profiles signify greater rizatriptan permeation from film F1 as compared to other formulations tested. In the case of film F1, the lag time was 0.52 ± 0.07 h, while it was 0.66 ± 0.11 h and 0.73 ± 0.15 h in film F4 and the control, respectively. The steady state flux value displayed by optimized film F1 (71.94 ± 8.26 µg/cm^2^/h) was more statistically significant (*p* < 0.005) than F4 (52.80 ± 6.05 µg/cm^2^/h) and the control (28.08 ± 5.02 µg/cm^2^/h). Similarly, the total quantity of rizatriptan transported into the receiver fluid at 6 h was statistically significant (*p* < 0.005) in films F1 (427.76 ± 51.69 µg/cm^2^) and F4 (323.22 ± 33.94 µg/cm^2^) when compared to the control (177.44 ± 37.78 µg/cm^2^). The permeability coefficient was also higher in film F1 (1.12 × 10^−3^ cm/h) than film F4 (8.25 × 10^−4^ cm/h) and the control (4.39× 10^−4^ cm/h). The enhancements in permeation were ~2.6- and 1.9-fold higher in films F1 and F4, respectively, when compared with the control. The greater permeation rate observed in film F1 may be allied to its greater drug release, seen in Figure 5. As the drug permeation was relatively higher in film F1, it was chosen for further in vivo investigation.

### 3.8. In Vivo

**Figure 7:**
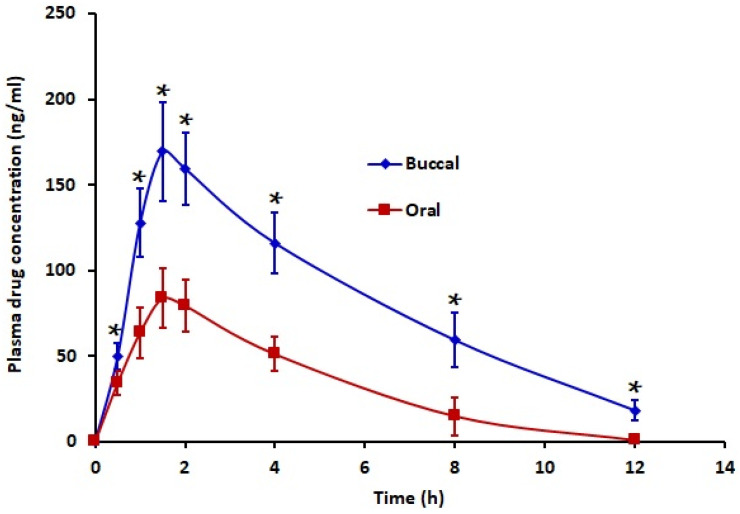
Mean plasma concentration–time profile of selected buccal film (F1) and control (oral solution of rizatriptan equivalent to 10 mg) in rabbits. Data represented are mean ± SD (*n* = 6). * Statistically different at *p* < 0.005.

Animal experiments were performed in the final stage of the study to assess the in vivo performance of the selected film (F1) compared with an oral solution. The plasma drug concentration–time profiles obtained after a single buccal administration of film (F1) and an oral solution of rizatriptan are illustrated in Figure 7, and the pharmacokinetic parameters determined are summarized in Table 4. It is obvious from Figure 7 that the rizatriptan plasma level was significantly high (*p* < 0.005) upon buccal film application throughout the study period (up to 12 h) as compared to the oral solution. In both cases, the absorption was rapid, as evidenced by high plasma drug levels detected within 30 min (buccal, 49.82 ± 7.96 ng/mL; oral, 34.32 ± 6.76 ng/mL), though statistically significant (*p* < 0.005). Buccal administration exhibited an increased C_max_ value (169.43 ± 28.67 ng/mL), which was approximately two-fold higher (*p* < 0.0001) than its oral counterpart. A rapid decline in drug plasma level was noticed in both treatments after the C_max_, most likely due to the short half-life (2–3 h) of rizatriptan. Being a BCS class III drug, the intrinsic permeability of rizatriptan was likely to be low, which was evidenced by the low AUC values in both treatments (Table 4). However, the observed AUC_0–12h_ in rizatriptan film was statistically significant (*p* < 0.0001) and was two-fold higher than the oral administration. Compared to oral therapy, the relative bioavailability judged from the AUC_0–12h_ of the average profile was approximately 244.77%. The increased rizatriptan level in buccal therapy indicated sufficient permeability of drug via the buccal mucosa. However, oral therapy of rizatriptan generally undergoes extensive first pass metabolism in the liver, thus causing a reduced drug plasma level compared to the buccal route. On the other hand, the same T_max_ value (1.5 h) was noticed in both the oral and buccal routes (Table 4).

## 4. Conclusions

In the current study, we investigated the practicability of developing a rizatriptan buccal delivery system in order to improve its clinical efficacy in migraine and to minimize adverse drug reactions. Buccal films loaded with rizatriptan (F1–F4) were successfully prepared using Proloc, HPMCs and Eudragit RS 100. All the developed buccal films exhibited optimal physicomechanical and pharmaceutical characteristics. In vitro release data displayed greater and complete drug release from film F1. Ex vivo data demonstrated greater rizatriptan permeation by film F1. The drug particles embedded in film F1 were compatible with the polymer used and demonstrated suitable characteristics for buccal application. The significant increases in drug plasma levels and C_max_ upon buccal film application indicated improvement in the extent of rizatriptan absorption and thus could be a valid means to enhance its clinical efficacy. Overall, the data in this study demonstrate that buccal therapy of rizatriptan can be considered as a potential and feasible approach for its successful treatment for migraine in the near future.

## Figures and Tables

**Table 1:** Compositions used for preparing placebo films.

Batch Code	Proloc (% *w*/*v*)	HPMC F4M (% *w*/*v*)	HPMC K100M (% *w*/*v*)	HPMC K4M (% *w*/*v*)	Eudragit RS 100 (% *w*/*v*)	PEG 200 (% *w*/*v*)	PG (% *w*/*v*)	Tween 80 (% *w*/*v*)	Film Properties
FR1	10	-	-	-	15	2.5	2.5	0.5	Non-homogenous film formed after drying
FR2	8	2	-	-	15	2.5	2.5	0.5	Small cracking occurred after drying
FR3	6	4	-	-	15	2.5	2.5	0.5	Films formed were non-tacky, peelable and possessed enough mechanical strength
FR4	4	6	-	-	15	2.5	2.5	0.5	Films formed were non-tacky, peelable and possessed enough mechanical strength
FR5	8	-	2	-	15	2.5	2.5	0.5	Films formed were non-tacky, peelable and possessed enough mechanical strength
FR6	6	-	4	-	15	2.5	2.5	0.5	Films formed were sticky
FR7	4	-	6	-	15	2.5	2.5	0.5	Films formed were thick
FR8	8	-	-	2	15	2.5	2.5	0.5	Small cracking occurred after drying
FR9	6	-	-	4	15	2.5	2.5	0.5	Films formed were non-tacky, peelable and possessed enough mechanical strength
FR10	4	-	-	6	15	2.5	2.5	0.5	Films formed were sticky

HPMC: hydroxypropyl methylcellulose; PEG: polyethylene glycol; PG: propylene glycol.

**Table 2:** Compositions used for preparing rizatriptan containing buccal films.

Batch Code	Rizatriptan (% *w*/*v*)	Proloc 15 (% *w*/*v*)	HPMC F4M (% *w*/*v*)	HPMC K100M (% *w*/*v*)	HPMC K4M (% *w*/*v*)	Eudragit RS 100 (% *w*/*v*)	PEG 200 (% *w*/*v*)	PG (% *w*/*v*)	Tween 80 (% *w*/*v*)
F1	6.4	6	4	-	-	15	2.5	2.5	0.5
F2	6.4	4	6	-	-	15	2.5	2.5	0.5
F3	6.4	8	-	2	-	15	2.5	2.5	0.5
F4	6.4	6	-	-	4	15	2.5	2.5	0.5

HPMC: hydroxypropyl methylcellulose; PEG: polyethylene glycol; PG: propylene glycol.

**Table 3:** Characteristics of prepared rizatriptan buccal films.

Batch Code	Thickness (Mm)	pH	Folding Endurance (Number)	Drug Content (%)	Mucoadhesive Strength (N)
F1	1.24 ± 0.27	7.2 ± 0.3	295 ± 20	95.1 ± 3.6	7.0 ± 0.4
F2	1.02 ± 0.16	7.0 ± 0.1	270 ± 18	94.8 ± 2.7	6.5 ± 0.3
F3	1.32 ± 0.34	6.9 ± 0.2	305 ± 26	96.0 ± 1.8	7.3 ± 0.2
F4	1.18 ± 0.21	7.1 ± 0.3	285 ± 15	94.7 ± 3.1	6.7 ± 0.3

All values are expressed as mean ± S.D; *n* = 6.

**Table 4:** Pharmacokinetic parameters of rizatriptan in selected buccal film (F1) and control (oral solution of rizatriptan equivalent to 10 mg) in rabbits.

Parameter	Buccal Film (F1)	Control
T_max_ (h)	1.5	1.5
C_max_ (ng/mL)	169.43 ± 28.67	83.85 ± 17.35
AUC_0–12_ (ng.h/mL)	994.86 ± 95.79	406.45 ± 61.08

All values are expressed as mean ± S.D; *n* = 6.

## Data Availability

The data presented in this study are contained within the article.
